# SENSE OF AESTHETICS AMONG OLDER ADULTS NEW TO LONG-TERM CARE SERVICES AND SUPPORTS

**DOI:** 10.1093/geroni/igae098.3001

**Published:** 2024-12-31

**Authors:** Charlotte Weiss, Karen Hirschman, Mary Naylor

**Affiliations:** University of Pennsylvania, Philadelphia, Pennsylvania, United States; University of Pennsylvania, Philadelphia, Pennsylvania, United States; University of Pennsylvania, Philadelphia, Pennsylvania, United States

## Abstract

Engagement in aesthetic experiences and activities can contribute to older adults’ quality of life and social connection. Few studies explore older adults’ sense of aesthetics (the extent to which one obtains pleasure from sensory awareness and appreciation of beauty) and how this information may be useful in supporting older adults during transitions to long-term services and supports (LTSS; in home, assisted living, or nursing home). We conducted a secondary analysis of interviews with older adults new to LTSS (R01AG025524) to examine the factors associated with Sense of Aesthetics (SOA) [a Dementia Quality of Life subscale]. A total of 376 LTSS recipients had complete data. The average score of the SOA was 3.44 (SD: 0.98, range= 1-5, median=4; higher scores indicate a greater sense of aesthetics. Using multivariable linear regression, we controlled for the influence of important health-related quality of life covariates (e.g., LTSS setting, sex, age, education, number of comorbidities, sensory impairment [vision/hearing], cognitive status). Older adults with greater positive interaction with social support (p>0.001) and a greater spiritual/religious disposition (p=0.004) had higher SOA. Older adults receiving LTSS in the nursing home had lower SOA compared to those in the home (p=0.029) and no difference between those receiving LTSS in home vs assisted living (p=0.49). The findings highlight the opportunity for LTSS intradisciplinary teams to consider the value that older adults assign to aesthetics and increase access to aesthetic experiences, especially within the nursing home setting. Implications for innovative ways to increase older adults’ sense of aesthetics will be discussed.

